# PD67 Subsidy Of Monoclonal Antibodies In Brazil: What Criteria Influence Decisions by the National Committee for Health Technology Incorporation (CONITEC)?

**DOI:** 10.1017/S0266462325101992

**Published:** 2025-12-29

**Authors:** André Motta-Santos, Daniel Reis, Júlia Tupinambás, Lélia Carvalho, Karina Zocrato, Marcela Freitas, Marcus Borin, Maria Horta, Clarice Petramale, Silvana Kelles, Sergio Bersan

## Abstract

**Introduction:**

Over the years many researchers have sought to understand the decision-making process of CONITEC, the Brazilian health technology assessment (HTA) agency. The emergence of high-cost technologies, such as monoclonal antibodies (mAbs), has heightened the importance of delineating the decision-making criteria. This study aimed to elucidate the criteria employed by CONITEC for the incorporation of mAbs in Brazil.

**Methods:**

All CONITEC reports published between 2019 and 2024 on mAbs were included. Descriptive statistics were utilized to summarize the data. The statistical significance between outcomes and covariates was assessed using the Kruskal-Wallis test for continuous variables and the chi-square test for categorical variables. Logistic regressions were produced to evaluate the impact of each covariable on the recommendations. Results with a p-value of less than 0.10 were considered statistically significant due to the small sample size. All analyses were conducted using R software.

**Results:**

After cleaning the database, 53 reports were included, encompassing 37 mAbs evaluated for 45 indications. Sixteen submissions (30.1%) received a positive recommendation. Efficacy (p=0.009) and the preliminary decisions (p<0.001) were significantly associated with the final recommendation. Further analysis showed that an incremental cost-effectiveness ratio (ICER) below BRL150,000 (USD65,020) was associated with a higher chance of a positive recommendation (p=0.050). Logistic regression revealed that the logarithm of the ICER was significantly associated with recommendations (odds ratio 0.618; p=0.031), indicating that increasing the ICER by 2.7 times was associated with a 38.2 percent lower chance of listing.

**Conclusions:**

Efficacy is important for decision-makers in Brazil. However, the results of economic analyses also influence the recommendation of mAbs. An ICER above BRL150,000 (USD65,020) per quality-adjusted life year significantly reduced the chances of a mAb being listed.

